# Glasgow Royal Infirmary

**Published:** 1830-04-01

**Authors:** 


### ROYAL INFIRMARY OF GLASGOW.

#### Tumours of the Female Breast.*

The surgeons of the Glasgow Infirmary deserve credit for their zeal in continuing the publication of such interesting cases as occur in the establishment. The present report is from the pen and practice of Mr. Cowan, who, we suppose, has recently succeeded to the office of surgeon, which is only held for a certain length of time by one individual. We shall select, as usual, the most interesting points for our readers' perusal, and none can be more so than the affections of the female breast. The lectures of Sir Astley Cooper have done much, and the splendid work he is now engaged in promises to do more in forwarding our acquaintance with these very important diseases. But to consider more particularly the present cases.

Case.—*The Irritable Breast.* "A young woman, about twenty years of age, of a strumous habit, admitted with irritable tumour in each breast. The menstrual secretion was irregular, and about the time of its appearance, the breasts became more irritable. Leeches had been applied formerly with little or no benefit. In the treatment of this patient, the only local application made use of was a plaister, composed of soap cerate, and extract of belladonna, and the whole attention was directed to the improvement of the constitution. Plum-

* Glasgow Journ. No. VIII. Vol. II.

ner's pills, along with decoction of sarsaparilla, were first employed, and laterally the mist. ferri comp. and other tonics. From these remedies, some partial amendment took place, and she was after some weeks sent down to the sea-coast. In such instances we must not be too liberal in the use of leeches, as their frequent repetition increases that irritable state of the constitution on which the existence of the tumour generally depends. The disease generally abates as the patient gets older."

We perfectly agree with Mr. Cowan in his remarks on the foregoing case. Cæteris paribus, the less local treatment employed the better for these irritable, or more properly speaking, hysterical, affections of the breast, or indeed of any other part of the body. They tend to direct the attention of the patient to a subject from which it should be the practitioner's object to wean her, and leeches or cupping-glasses in these cases not unfrequently "make the food they feed on." We remember, some few months ago, having been particularly struck with this fact, in the instance of a young woman labouring under the hysterical affection of the elbow-joint. A surgeon had treated it by caustic issues, and the patient became worse. Another surgeon saw the girl, and prescribed bread pills and aloes and myrrh, but not effecting a cure in the course of a week, or even less, he, too, became alarmed, abandoned his former scent, although the true one, and followed in the wake of his predecessor by again establishing a caustic issue. It was many months before the complaint wore itself out under this treatment, whereas, under the firmer use of antispasmodics, emmenagogues, and tonics, it would probably have been got under in as many weeks. These sentiments are not delivered at random; we speak from experience, and that by no means a narrow one. When the pain is very obstinate or very severe, which it not unfrequently is, we think, on the whole, that blisters answer better than either leeches, cupping, or any other method. At the same time, we always have recourse to these measures with reluctance, for we really think it an unjustifiable thing to disfigure and score the neck, breast, and back of young women, for complaints that, in nine cases out of ten, would do equally well, or better, without any local treatment at all. Purgatives are generally of essential service, but they should almost always be combined with aromatics or tonics. However, this is such an interesting subject, that were we once to embark in it, we should far exceed the limits of this article. We, therefore, pass on to the consideration of a much more serious disease—cancer.

"Three cases of cancer were admitted: in one of these, a patient of forty years of age, married, with a family, the complaint was only of seven months' duration, but the whole breast was implicated in the disease; the tumour was ulcerated, and firmly attached to the subjacent parts; the glands in the axilla were enlarged, and the lung seemed diseased, dyspnœa and cough being present. This case being entirely hopeless, the patient was dismissed.

An old woman, nearly seventy, had distinct scirrhous tubercle in the left breast, but this gave her little uneasiness, unless from the mental distress it occasioned. From the slow progress of similar tumours at an advanced period of life, she was advised to dismiss all uneasiness from her mind regarding it, and to allow no application whatever to be made to it. She was dismissed relieved.

The other case was that of a married woman, the mother of several children.

In the left breast is a very hard tumour, occupying more than two-thirds of its substance, and about the size of half an orange. The tumour has an irregular and slightly nodulated surface, and the integuments at its outer margin are retracted and adherent to it. It moves freely over the subjacent parts; is the seat of frequent lancinating pain, which darts through it into the axilla, where several indurated glands are felt, the two largest the size of a hazel nut. Slight cough towards evening. Dyspepsia: pulse 76. Leucorrhœal discharge, but on examination, no disease of the uterus can be detected.

Swelling was first observed four years ago, and was nearly of the size of a pea. In a year it attained the size of a hazel nut, and became affected with lancinating pains, which have increased in severity with the size of the tumour. Pain first felt in the axilla three weeks ago, and within the last few days, the indurated glands were first observed.

20th March. The mamma, and the indurated axillary glands, were removed in the usual manner; and when examined, the tumour was found to be distinctly scirrhous. The wound was dressed on the 24th, and appeared healthy; the greater number of the ligatures having come away. On the 26th, the remaining ones were detached—the discharge was copious and the lips of the wound were inflamed. She complained of cough and pain in the chest, increased on coughing. She was bled to ℥xij. She rapidly improved: the dyspeptic symptoms yielded to tincture of colomba and quinine. She recovered her strength and flesh, and on the 21st April, a month after the operation, she was dismissed cured."

The subject of the next case was an unmarried female, aged 37. On the date of report, the 3d of December, the following were the symptoms:—On the right side of the chest, extending downwards over the mamma, from the junction of the first rib with the sternum, a deep ulcerated cavity, about five inches in length and three in breadth—edges of the sore everted—base florid and apparently not unhealthy—discharge thin, fetid, and considerable. The superior part of the sternum formed the inner boundary of the sore for upwards of two inches, and the bone at this point seemed projecting forwards. Below this the integuments were swollen and red, the whole mamma was also swollen, firm, and painful upon pressure. A little above the ensiform cartilage was a small foul sore, the size of a sixpence, with everted edges. One of the axillary glands was as large as an orange, and several glands above the clavicle were indurated. She had frequent pain in the sore and the axilla, stretching up to the neck—occasional cough and tendency to dyspnœa—catamenia present every three weeks—general health otherwise tolerable.

A small tumour first appeared four years before the date of report, and increased so slowly, that ten months back it was only the size of a child's fist. It then ulcerated, and subsequently the ulceration progressively increased. Her mouth was made sore with mercury, and a solution of chloride of lime employed to destroy the fœtor of the discharge, whilst the breast was supported and the sore simply dressed. For a week previous to the 22d, very firm pressure by compress and roller was made upon the tumour; but under this treatment matters became worse, and it was, therefore, discontinued. On the 5th of January she was ordered quinine and wine, and this was followed by a favourable change in the appearance of the sore. By a mistake, mercurial pills were now substituted for the quinine, the mouth became affected, and the ulcerated surface very irritable and sloughy. Bloody discharge ensued, then repeated hæmorrhage, the pleuræ were involved in the sloughing and laid bare, and on the first of February the patient died.

*Sectio Cadaveris.* "The ulcer had extended itself so as to occupy more than the superior two-thirds of the right side of chest; was universally of a dark gray colour and sloughy appearance.

In the axilla, and in the seat of the induration which existed in that quarter, sloughing had taken place to such a depth as to expose the axillary vessels and nerves. The sore thus produced was about two inches distant from that on the breast. Towards the sternal boundary of the latter sore, the pleura was perforated at several points, and the cartilages of all the ribs on the right side but the first were loosened, and some of them completely detached from the sternum.

All along the line of its attachment to the cartilages, and extending some way over its anterior surface, this bone was found rough. In the situation of its junction with the cartilage of the second rib, the upper and middle portions of the sternum were nearly separated from each other, and about an inch and a half of its inferior extremity was carious, and completely detached from the middle portion.

On the left side, the cartilaginous attachments of the 2d, 4th, 5th, 6th, and 7th ribs to the sternum were much loosened. The right lung was partially adherent to the side of the chest, its surface coated with flakes of coagulable lymph, and its substance partially consolidated from recent inflammation.

Several ounces of sero-purulent fluid were found in the right side of the chest. On the left were some adhesions, but the lung was healthy. The left mamma was enlarged and indurated, but presented no fibrous bands.

From the patient's age, general health, and the absence of symptoms denoting cancer, but especially from the purulent nature of the discharge, and the very great and decided improvement produced by the tonic mode of treatment, I am convinced that the above case was not one of cancer, and until the lamentable mistake of her taking mercurial for quinine pills, I had every prospect of seeing this large ulcer healed. The disease of the bones found on dissection was not greater than the phagedenic nature of the sore latterly was calculated to produce, and it is barely possible, that had cancerous action been going on in the tumour for years, that the viscera would have been so healthy as on inspection they were found."

We perfectly concur with our author in not considering the above as a case of cancer, but rather as one of unhealthy or phagedænic ulceration, produced perhaps by causes of which both he and we are ignorant. We cannot but lament the substitution of the mercurial for the quinine pills, and yet we are scarcely so sanguine as our author with respect to the ultimate result had no such mistake been committed. We pass on to a case of the hydatid tumour of the breast.

*Case.* J. C. æt. 60, presents, April 3d, a tumour of large dimensions situated over the left side of the chest, fluctuating below, firm in its upper fourth, and irregular on its surface. It measures round its base two feet one inch; increasing as it proceeds outwards, its greatest circumference is two feet four inches; across its summit in one direction it is one foot five, in another one foot two inches. In the axilla is a gland the size of a walnut, and below this an induration felt as if prolonged from the tumour, extending up the latissimus dorsi, and moving pretty freely on the subjacent parts. Complains of sharp pains shooting occasionally through the tumour and most severe at its lower part—pulse 76—appetite much fallen off—countenance anxious and sunk—cough with slight and not purulent expectoration—dyspnœa after exertion. A year ago first observed a hard tumour not larger than a pigeon's egg in the mamma. It was unattended by pain, and did not enlarge much for three months when she struck it; a thin discharge from the nipple ensued and continued for the space of three weeks, after which the tumour became affected with sharp pain and increased with more rapidity. The only remedies she has used were frictions with turpentine and spt. camphor.

"On the 9th, a puncture was made with an abscess lancet in the most depending part of the tumour, and about lbs. iv. of fluid, of a dark bloody colour, evacuated. The tumour was then dissected out in the usual way. Besides the large cyst, the contents of which had been evacuated, four others of a smaller size were found; they were all firmly connected together, and to the subjacent parts, by a fibrinous substance, chiefly semi-transparent, and nearly colourless. In the lesser cysts, the fluid was without colour; in the large one, as has been already said, it was bloody, and attached to the walls of this cyst; there was also a quantity of thick soft pasty matter, of a light brown colour, and separable only by maceration. Every diseased part, however, was carefully dissected out. Although no unusual quantity of blood was lost; the incision was necessarily of immense extent, and the patient seemed so exceedingly exhausted, that a general impression prevailed that she would not survive the night. Fifty drops of laudanum, and ℥ii. of brandy, were given immediately after the operation, part of which was vomited. At bed-time she had gr. ii. of opium, and passed a good night."

On the 12th the wound was found adherent, and on the 9th of May the patient was discharged with the wound cicatrized, the appetite excellent, and cough trifling.

We suppose this must be considered a tolerably fair specimen of the hydatid tumour of the breast, though it does not tally with the ordinary appearances of that disease in every particular. We are on the whole disposed to think that this "hydatid" disease is not separated from the medullary fungus and scirrhus, by such broad and bold lines of demarcation as is commonly imagined. Certain it is that both in scirrhus and fungus hæmatodes, especially in the latter, we do very frequently find hydatids, or rather cysts filled with dark coloured serum, resembling, apparently in every respect, the larger ones that constitute the peculiar hydatid disease of the breast, or other part. Happily experience, our great instructor, has shewn us that whether it be allied to the more malignant affections or not, the "hydatid" tumour is much less likely to return after operation than they are, in other words that its grade of malignancy is less. This is the cheering and practical point. It is equally surprizing and gratifying to find that these large tumours of the breast may be safely removed at a very advanced period of life. The present patient was sixty, and in another case in which we saw the operation successfully performed by Mr. Brodie at St. George's Hospital, the individual was seventy-five years of age. Mr. Brodie, if we remember right, remarked on that occasion that he had removed several such breasts, and the patients had survived for many years afterwards without any return of the disease.

An interesting case of fungus hæmatodes is related, but this we pass over for the present, as we will probably make it the text of a short article at another opportunity.

### ROYAL INFIRMARY OF GLASGOW.

### LOOSE CARTILAGES IN THE KNEE-JOINT.*

*Case.* D. M. ætat. 24, farm-servant, complains, Dec. 3d, that the motions of his right leg are much impeded by some firm moveable bodies in the cavity of the knee-joint. He thinks they are three in number, but has never been able to fix more than two of them at one time; the largest is the size of a hazel nut, the smallest that of a field bean. No increased secretion of synovia nor inflammation of the joint. States that two years ago he first felt uneasiness in the joint when at work, and twelve months afterwards observed a loose body moving in the joint. This was removed five months ago, and when he recovered from the operation the present ones were discovered. On the 7th, the substances being previously secured at the inside of the joint, and an assistant having drawn the integuments outwards, an incision was made at once through the integuments and capsule of the joint, and the two substances easily extracted.

The lips of the wound were brought together by plaster and bandage, and motion effectually prevented by a splint placed under the limb. On the 12th the wound was firmly healed, and on the 29th the patient dismissed cured. On examining the substances removed from the joint they appeared externally to be entirely cartilaginous, but on making a section of each the smaller was found to be nearly equally composed of cartilage and bone, the larger much more completely ossified.

"It has been stated that the existence of such bodies is generally accompanied with increased secretion of synovial fluid. In some of the first cases of this description, particularly in that given by Paré, a very considerable effusion into the knee-joint had taken place, and the operation of opening the joint was performed to evacuate the fluid, and during this, the foreign body presented itself at the opening, and was extracted. In general, the discovery of such substances has been preceded by injuries of the joint and slight inflammatory action; but in others, sudden lameness takes place, and examination of the joint discovers the cause, without any well marked inflammation.

"With respect to the substance of which they are composed, I believe them all to be primarily effused lymph, but in process of time they become cartilaginous, ossified in part, and ultimately wholly so. In repeated instances have such bodies extracted from the same knee presented all these characters, thus proving that the differ-

* Glasgow Journal, No. VIII.

ence of texture depended solely on their duration in the joint.

"Some have regarded these substances as pieces of cartilage, detached by falls or bruises from the articulating surfaces of the joint; while others, and with more reason, consider them as arising quite independent of this cause.

"Monro, Baillie, Richter, Wardrop, and others, have found similar substances, cartilaginous and osseous, in the tunica vaginalis testis, complicated with hydrocele; and one example of this kind came under my own observation. In some of these instances, small portions of coagulable lymph were likewise found.

"I have had no opportunity of examining the knee joint of any person who died with such substances in the joint, nor am I aware of any such dissection being on record; but judging from the appearances in the coverings of the testicle, when similar bodies are found there, I should be inclined to expect that where they form in the knee or other joints, they are the result of inflammatory action, producing effusion of lymph, which passes through the cartilaginous state into the osseous. The capsular ligament, when the disease has continued long, will, in all probability, be thickened.

"The old axiom, that wounds of joints if not mortal are exceedingly dangerous, has still influence over the minds of some practitioners, and induces them to recommend a palliative treatment by means of bandages, to removing them by an incision.

"When, however, no inflammatory action is present in the joint, when the patient is young and healthy, and means are taken to prevent all motion of the joint after the operation, I conceive the extraction of these bodies as by no means likely to be attended with danger. There are some constitutions, however, in which the most trifling operation is likely to prove dangerous; and I once saw most alarming symptoms follow the operation, attributable, however, in some degree, to the patient having used the limb soon after the operation. It is always proper to put this out of his power, by means of splints."

We are disposed to agree with Mr. Cowan in believing that this operation has been regarded with a too superstitious dread by the older surgeons. We must take care however lest we run into an opposite error, and exchange the extreme of caution for temerity, an exchange which would certainly be for the worse. Some patients have died after the operation for removing loose cartilages from the knee-joint, and more have been placed in imminent peril. What all the world says may not be strictly true, but it never is entirely false, and all the world have pronounced that a wound of a large joint is not to be looked on as a bagatelle. We grant that bad consequences seldom ensue except in a person of weak habit or injured constitution. But this kind of argument is not worth so much as might be thought, for the evidences of that bad habit and constitution are by no means always apparent before the operation, and consequently the surgeon only ascertains it by the result. Of this we have seen more than one example. With regard to the application of splints, we can bear our testimony to the advantages derived from their employment. If perfect repose is procured for the joint and vigilant caution exercised in guarding against inflammation, we believe that loose cartilages may be extracted from the articulation with much *less* danger than is commonly believed. For the mention of one or two interesting cases we may refer the reader to the 20th Number of this Journal, page 524.

### GLASGOW ROYAL INFIRMARY.*

Our Glasgow contemporary, we see, has changed masters, Mr. Mackenzie retiring from the helm, and Drs. Buchanan and Weir stepping into the vacant post. We trust that the alteration will not be for the worse, and that a journal from which we have derived much information, and which has always been conducted in a tone of proper and gentlemanly feeling, will not be suffered to languish and die. The reasons for the change of editors we do not profess to know, though the sign is generally considered an ominous one in the history of medical periodicals. Whatever it is, we beg to offer our best regards to Mr. Mackenzie on his ceasing to be one of our confréres.

I. FRACTURES, SIMPLE AND COMPOUND.

"The fractures of the thigh and leg were all put up in the straight position, with Dessault's splints and bran pillows, as usual at this hospital, and with one exception, were all cured without the slightest shortening or distortion of the limb. In that case the bone was obliquely fractured immediately below the trochanter: the man insisted on leaving the house before union was quite perfect, and he fractured the same thigh a second time, and afterwards could not bear the necessary extension. In the case of a feeble old man of 78, the bone was found united in 46 days, and he was dismissed soon after without the slightest shortening, although the fracture was oblique and in the upper third. In another case, a stout Englishman, aged 50, a china hawker, previously in good health, the fracture occurred immediately above the condyles, and was occasioned by strong muscular exertion, while endeavouring to raise a heavy basket of china. It was united at the end of eight weeks. A fourth case, was a very severe comminuted fracture about the middle of the thigh. A cart heavily loaded passed over the limb, and besides breaking the bone, produced considerable bruising of the soft parts and a small wound of the integuments. This was sent in as a very bad case, but the limb was immediately put up, and union took place without any accident in 44 days. In the other 3 cases, one of which was a boy aged 2½ years, the bone was broken about its middle, and nothing unusual occurred.

"The two cases of fractured fibula were put up with a single splint on the inside of the leg, as recommended and practised by Dupuytren, a figure of eight bandage being applied round the ankle. This method appears to me well adapted to prevent the lateral displacement of the inferior end of the bone, so apt to take place when the fracture occurs, as it generally does immediately above the external malleolus."

In a case of rather severe compound fracture of both bones of the left leg about three inches above the ankle-joint, dry lint was applied, the leg put up in splints, and the dressings, &c. left undisturbed for *five weeks*, when merely a healthy looking superficial ulcer of the integuments remained. In the course of another week the sore was cicatrized. A case of compound fracture of the olecranon proved fatal.

*Case*:—M. M. æt. 46, drunkard, of bad constitution, was admitted Sept. 4, having fallen in the street, and fractured the neck of the humerus and the olecranon transversely; wound of the integuments leading to the fracture—much swelling of the limb—considerable effusion into the elbow-joint. Leeches &c. were applied, the swelling subsided, but profuse suppuration took place, communicating with the joint, and extending nearly to the axilla and

---

* Glasg. Journ. No. IX.

some way down the fore-arm. Two loose portions of bone were removed, wine and spirits with opium and carbonate of ammonia were liberally given, but she sank under the irritation and wasting discharge. Sloughing had taken place to a great extent along the outside of the arm, a free opening into the elbow-joint existed which was much diseased, the integuments were undermined as high up as the scapula, and the cellular membrane was sloughy throughout, the fracture of the neck of the humerus was nearly united, and no disease existed in the chest or abdomen. The two following cases are interesting; the first appearing to Dr. Weir to illustrate in a striking manner, the good effects of the practice of little interference after the dressings are properly applied.

*Case.* "J. M'A. aged 65, was knocked down by a carriage, the hind wheel of which passed over left leg, and fractured both bones about three inches above ankle joint. The fracture of the fibula was comminuted, and there was a wound of the integuments, close upon the inner edge of the tibia, through which the probe could be passed to the fractured ends of the bone, and also under the integuments for some inches in every direction. There had been considerable hæmorrhage from the wound, and much bruising and swelling of the whole limb. The leg was immediately put up in splints, dry lint being applied to the wound. These were removed for the first time at the end of *five weeks*, when the fracture was found united, and there remained only a superficial healthy looking ulcer of the integuments. The splints were again applied for another week, and then finally removed, when the sore was found cicatrized. He never had a bad symptom, and was dismissed in good health. In this case, had the wound been left exposed, and leeching and warm poultices had recourse to, which I have seen done in similar accidents, I have no doubt that great inflammation and suppuration would have taken place, and the leg probably lost, or if not, a tedious cure would have been the consequence.

"In a second case, where the wound of the soft parts appears to have been originally as trifling, amputation was required at the second period, and the man recovered contrary to expectation.

"A. P. aged 50, was admitted on the 1st September, with a transverse fracture of the left tibia, two and a half inches above the ankle joint, and a severe comminuted fracture of the fibula of the same leg. The accident was produced by the wheel of a loaded cart passing over the limb seven days before admission. There was a small wound of the integuments discharging thin unhealthy matter, and through which the probe passed to the fractured ends of the tibia. The whole limb was very much swollen, the integuments of a red colour, and vesications existed on its lower third, apparently produced by irregular bandaging previous to admission. His general health was bad. He laboured under pneumonia, and had been subject for many years to cough and dyspnœa, with occasional hæmoptysis. These complaints had been increasing for some months previous to the accident. He lost thirty ounces of blood at two bleedings, which was buffy and much cupped; the chest was blistered, and he was put upon calomel and opium; the wound was dressed with turpentine liniment, and the discharge being profuse, the dressings required to be removed every three or four days. The pectoral inflammation subsided in about a week, but the wound in the leg enlarged gradually, and in a few days it became sloughy, and the superior fragment of the tibia was exposed denuded to the extent of several inches. His strength now fell off rapidly, and the limb was removed on the 19th September. The fracture of the tibia was found to have extended into the ankle joint, the inferior fragment having a fracture running nearly perpendicularly through it. The joint contained a quantity of fetid pus, and the cartilages were in some points destroyed. The fracture of the fibula was much comminuted, and several portions of it were wholly detached, and lying loose among the integuments. The pulse fell immediately after the operation; his appetite became keen; his pectoral complaints did not return; and with the assistance of quinine and a nourishing diet, he rapidly recovered, and was dismissed, on 1st November, in perfect health."

The latter is a very fortunate and a very instructive case. It has been remarked by the most experienced surgeons, at least we have heard Mr. Brodie make the observation, that when patients are labouring under diseases of the joints and symptoms of thoracic disease, the latter will occasionally be removed by the removal of the former. On this principle we have seen Mr. Brodie perform amputation for diseased joint, when the patient had most of the symptoms of phthisis, and in one or two cases the issue was successful. The present instance would seem to be one of the same kind, and is calculated, with what we have mentioned, to encourage surgeons not to abandon a patient as always improper for an operation, because the thoracic organs may appear to be affected.

II. Delirium Tremens.

The attention directed to this remarkable disease of late years has been productive of excellent effects, by setting surgeons and physicians on their guard, in the use of the lancet and antiphlogistic measures. In this the blindest may perceive the advantages arising from the cultivation of periodical literature, the medical journals conveying the dicta of experience to those who have had no experience at all; and putting one man on his guard by the record of an error that has happened to a brother practitioner separated from him by many a mile, perhaps by the broad ocean, speaking another tongue, or habiting another hemisphere. Would it were for this alone that the medical periodical press is distinguished, for all might then be proud of wielding an engine of good. This, however, is an inglorious kind of fame, not coveted by the factious spirits of the day, who aspire only to hurl the brand of discord into the bosom of the profession, and reduce every member of it to the level of their own vices and buffooneries.

Their mouth is black as bull dog's at the stall;
They scratch and bite, and spare ne lace, ne band,
But bitch and rogue their answer is to all.

But away with the thoughts and the mention of these foul traducers, and let us pass once more to the pleasing occupation of receiving and conveying professional instruction.

*Case.* "P. C. a healthy man, aged 21, was brought into the hospital on the 18th September, having suffered a simple fracture of the left tibia a little above its middle. Two days after admission, he was seized with delirium tremens. At midnight he tore the splints and bandages from his leg, and walked about the ward, and it was necessary to strap him down to bed and put on a strait jacket. Next day the disease was well marked. Besides the usual symptoms of incoherency and constant jactitation, the great loquacity, the spectral illusions, and the total forgetfulness of the accident, symptoms so peculiar to this affection, were all distinctly present. Notwithstanding the liberal use of opium and spirits, to which he had been previously addicted, with the addition of shaving the head and keeping it constantly cool, the disease increased. One grain of opium was given every two hours, and laudanum was exhibited in the form of enema. About twenty-four hours from the commencement of the attack, after having been for a short time extremely violent, he fell into a profuse perspiration, and became rather more quiet, but rapidly sunk, and died within thirty hours from the first appearance of the delirium. This man never slept during the attack. On inspection there was a small quantity of serum found in the ventricles of the brain, and also at the base; there was no effusion beneath the membranes. The vessels of the pia mater were minutely injected with blood, and the substance of the brain, when cut into, presented very numerous bloody points. The brain was unusually firm, and altogether more vascular than natural."

Another case of delirium tremens, following a more severe injury, occurred at the infirmary during the last Summer. The disease was checked on the third day by opium and the use of spirits, to excesses in which the patient had been addicted. Our readers must be aware that M. Dupuytren, who has given a good description of this traumatic delirium, maintains that opium answers better when administered in enema than when taken by the mouth in the usual way. In the case to which we have just been alluding the correctness of the Baron's statement was exemplified, for the drug "whilst given by the mouth did not seem to produce much effect, but a full dose by the rectum was soon followed by a tranquil slumber," and rapid amendment ensued. The following remarks of Dr. Weir are very just.

"Delirium tremens is by no means an unfrequent disease. It is often seen in hospitals after injuries or operations, but many cases occur in confirmed drunkards without this complication. So far as I have observed, it usually continues from two to eight or ten days, although it is sometimes met with as a chronic affection. It is not often fatal. It very generally yields to opium, and this requires to be given in full and repeated doses, until sleep be procured. When this takes place all danger is generally at an end. Indeed the chief point in the treatment of this affection is to procure sleep. Although the opium and other means employed should produce a complete cessation from delirium, and the patient should become comparatively calm, coherent and collected, still if sleep does not take place, I believe very little good is accomplished. The disease will return again and again; opium therefore requires to be exhibited until this effect be produced, whatever may be the dose necessary. It has sometimes been given to the extent of a scruple in twenty-four hours, and with the best effects.

"From the general appearance of the patient in this peculiar affection of the nervous system, it has often been looked upon as an inflammatory affection of the brain, and treated with copious bleeding, brisk purging, blistering, &c. The lancet, however, is scarcely ever admissible, unless perhaps, in strong robust subjects who have not been much addicted to intemperate habits. I think I have seen bleeding adopted without any bad consequences following, although in the great majority of cases it will be hurtful. Some practitioners think that too much confidence is placed in opium in the cure of this affection, to the entire exclusion of every other remedy; but the result of the practice of giving large and repeated doses of this medicine, has been so very generally favourable that its efficacy is perfectly established. As to its *modus operandi*, or how it came to be employed in a disease where an opposite mode of treatment would appear to be so necessary, we have less to do, since the fact of its success is undoubted. It seems to produce its beneficial effects by soothing the excessively restless and irritable state of the patient, and by procuring sleep. That the disease is an affection of the brain and nervous system there can be little doubt, although the cases which end fatally do not in general exhibit any very well marked traces of organic derangement. From its sometimes ending in apoplexy or palsy, it may be supposed that there must exist some degree of fulness of the vessels of this organ, and there has sometimes been found, on dissection, serous effusion beneath the membranes and into the ventricles."

We can bear testimony to the excellent effects of opium in some of these cases of traumatic delirium. We lately witnessed a most remarkable instance of the affection in the person of a man who was much addicted to intemperance of all kinds, and who had met with a fracture of the bones of the leg. The case was considered by all to be a very bad one, and the quantities of gin and opium administered were very considerable indeed. The patient, who was in St. George's Hospital, eventually recovered, although he laboured under the delirium for *several weeks*, and an abscess formed in the cellular membrane about one shoulder. In another instance the exhibibition of opium was not so successful, gangrene of the fractured limb supervening and proving fatal.

### III. BURNS.

It is probably known that carded cotton is much used at the Glasgow Infirmary, in cases of scald or burn. It is the favourite application there to all recent burns. In the case of a collier, admitted on the 22nd of June, with his face, hands, and arms very much scorched from an explosion of fire damp, and the parts thickly coated with the coal dust, the whole was completely cicatrized in three weeks. The burn was severe on the face, but the cotton was changed once or twice only, and not the least scar was left. This man was delirious for some days after admission, and had many symptoms of approaching effusion into the brain, but these were checked by the liberal use of opium, which Dr. Weir has generally found of great service in affections of the head succeeding burns and scalds. In a case of extensive burn which proved fatal fourteen days after the accident the "posterior lobe" of both lungs was found partially consolidated by recent inflammation; the pleuræ were adherent, the mucous membrane of the bronchia red, and in several places covered with muco-purulent matter. The brain and abdominal viscera were healthy.

### IV. LUPUS TREATED BY THE IODIDE OF MERCURY.

"One very interesting case of this disease was in the Hospital for some months. I mention it here, because it has been greatly benefited by a medicine which is not yet much employed in this quarter; I mean the iodide of mercury.

"A. A. a healthy country girl, aged 21, had suffered under lupus for thirteen months at the time she was admitted. The tip and alæ of the nose were the parts affected, and there was a small portion of the cheek also involved, and the disease had in some degree spread to the inside of the nostrils. It presented in several places the distinct tuberculated appearance of lupus, and in other places had proceeded to ulceration, although this was not very deep. She had undergone a course of mercury, and used a great many local applications; among which were the nitrate of silver and a solution of arsenic, but with little permanent benefit. After her admission lunar caustic was repeatedly applied to the affected parts, and she used an ointment composed of mercury, camphor, and turpentine, and also a strong arsenical ointment, but not with much effect. The iodide of mercury, in the proportion of six grains and afterwards nine grains, to an ounce of simple ointment, was then used daily, and under this application many parts skinned over, and the disease was in a great measure subdued. She used it for several weeks in the Hospital, and since she was dismissed some of the small tubercles have re-appeared, but were speedily destroyed by the slight ulceration produced by the ointment. In this case, the disease, although not yet completely eradicated, has been kept under, and more completely relieved by this, than by any other application.

"I have also used the same preparation, but stronger, in a case of curious anomalous tumours of the face, somewhat resembling the melanosis, but evidently not malignant, although it could not prevent the re-appearance of the affection. I think this preparation of iodine and mercury will be found to answer in many cases of ulcerations where other escharotics, murias hydrarg. nitras argenti, arsenic, &c. have failed."

Here we must stop for the present, but some interesting cases remain to be noticed at a future opportunity.

---

